# The District Nurse

**Published:** 1916-11-04

**Authors:** 


*To the Editor of* The Hospital.

### The District Nurse.

*To the Editor of* The Hospital.

Sir,—In an article in The Hospital of 28th ult., entitled " District Nurses and their Work "—in trying to " indicate the different grades of nurses included " in " district nurses "—it states, " First there is the fully-trained Queen's nurse " and, lower down the page, " fully-trained " or " Queen's " district nurses. " Fully trained " and " Queen's " are not necessarily synonymous. There are many fully-trained district nurses who are not Queen's nurses. All Queen's nurses are not fully trained—*i.e.*, have not had three years' hospital or infirmary training. About ten or twelve years ago at the most, probably less, nurses were accepted on the " Queen's Roll " after two years' hospital and some district training. Since the Queen Victoria Jubilee Institute raised the standard to three years they did in some cases send some of their nurses to a hospital or infirmary to get another year's training afterwards. But, as everybody in the profession knows, this does not make them fully trained, which is three consecutive years in one training school of at least 100 beds and resident medical officer, and a certificate of training after. All Queen's nurses do not hold the C.M.B. certificate even, to say nothing of other qualifications, and yet get £100 inclusive! while people advertise for fully-trained nurses with C.M.B. certificate to do general district nursing, midwifery, health visiting, school visiting, tuberculosis visiting, etc., etc., and offer salaries of £75 to £85 inclusive! One I know has just been offered a post in a large, scattered, and hilly district, where cycling is impossible, and therefore a bicycle is not provided, and for the *inclusive* salary of £85 a year she is to do " the general nursing, midwifery, school work (*sic*), health visiting, notification of births, etc., as required by the County Council." She is to provide her own uniform, bag, etc.; and she may have a half-day once a month, *when work allows*! This is only one of many, and, although there are a few nurses who will accept such posts and " spoil the market," it is not surprising that district nursing is unpopular.

As to the remark in the article I have mentioned about the " social circumstances " of the " village nurse and Queen's nurse," nurses are taken from all classes, and the Queen's nurse is often no better socially than the village nurse. It might as well be said of the probationer in hospital that her " social circumstances " (?) " enable her to live more cheaply " than the staff nurse or sister!

All Queen's nurses are by no means well educated or of good social position, and to be a " Queen's " nurse gives one no status.—Yours faithfully,

Fully Trained Nurse and C.M.B.

